# Current FDA-Approved Therapies for High-Grade Malignant Gliomas

**DOI:** 10.3390/biomedicines9030324

**Published:** 2021-03-22

**Authors:** Jacob P. Fisher, David C. Adamson

**Affiliations:** 1 Division of Biochemistry, Southern Virginia University, Buena Vista, VA 24416, USA; 2 Department of Neurosurgery, School of Medicine, Emory University, Atlanta, GA 30322, USA; cory.adamson@emory.edu; 3 Atlanta VA Healthcare System, Decatur, GA 30033, USA

**Keywords:** high-grade glioma, malignant glioma, standard of care, glioblastoma, temozolomide, lomustine, carmustine, bevacizumab, tumor treatment fields, FDA-approved

## Abstract

The standard of care (SOC) for high-grade gliomas (HGG) is maximally safe surgical resection, followed by concurrent radiation therapy (RT) and temozolomide (TMZ) for 6 weeks, then adjuvant TMZ for 6 months. Before this SOC was established, glioblastoma (GBM) patients typically lived for less than one year after diagnosis, and no adjuvant chemotherapy had demonstrated significant survival benefits compared with radiation alone. In 2005, the Stupp et al. randomized controlled trial (RCT) on newly diagnosed GBM patients concluded that RT plus TMZ compared to RT alone significantly improved overall survival (OS) (14.6 vs. 12.1 months) and progression-free survival (PFS) at 6 months (PFS6) (53.9% vs. 36.4%). Outside of TMZ, there are four drugs and one device FDA-approved for the treatment of HGGs: lomustine, intravenous carmustine, carmustine wafer implants, bevacizumab (BVZ), and tumor treatment fields (TTFields). These treatments are now mainly used to treat recurrent HGGs and symptoms. TTFields is the only treatment that has been shown to improve OS (20.5 vs. 15.6 months) and PFS6 (56% vs. 37%) in comparison to the current SOC. TTFields is the newest addition to this list of FDA-approved treatments, but has not been universally accepted yet as part of SOC.

## 1. Introduction

Gliomas are brain tumors that originate from glial cells. They account for 28% of all primary brain tumors, yet they make up 80% of all malignant primary brain tumors in adults [1]. WHO Grade II gliomas are considered low-grade gliomas with more benign growth behavior and very long overall survival (OS), but recent molecular-based data have shown that some can have molecular features and growth patterns of high-grade gliomas (HGG); WHO Grade III and IV gliomas are categorized as HGGs and have a malignant growth pattern with very poor OS (Table 1). The most common WHO Grade III glioma is an anaplastic astrocytoma (AA). AA patients typically live 2–3 years after diagnosis. The most common WHO Grade IV glioma is glioblastoma multiforme (GBM). GBM is the most common malignant brain tumor, and makes up 45.6% of all malignant tumors [1]. GBM patients typically live 12–18 months after diagnosis. Anaplastic oligodendrogliomas (AO) are less common WHO Grade III gliomas.

The 2016 World Health Organization classification of gliomas (reviewed in [3]) is the most recent classification of HGGs [3]. This has greatly improved diagnosis and treatment recommendations as prior histologic classifications had overlapping tumor growth behaviors. For example, some WHO grade II gliomas appeared to have rates of progression similar to higher grades. Molecular changes, particularly in IDH, P53, ATRX, and 1p19q, have been pivotal in diagnosis and prognosis. For example, IDH-mutant and 1p19q-codeleted AO patients typically live 12–14 years. IDH-mutant GBM patients typically live 24–36 months compared to 12–18 months for IDH-wildtype GBM patients. IDH-1 wildtype AAs have similar tumor behavior and prognosis to GBM. These molecular characterizations are critical to better classify patients and more properly treat them. Gliomas with histology that resembles lower grades but have molecular features of higher grade gliomas will now get treated as higher grade gliomas. Given the genomic heterogeneity of gliomas, it is expected that future WHO classification schemes will continue to become more refined.

## 2. Standard of Care

**Figure 1:**
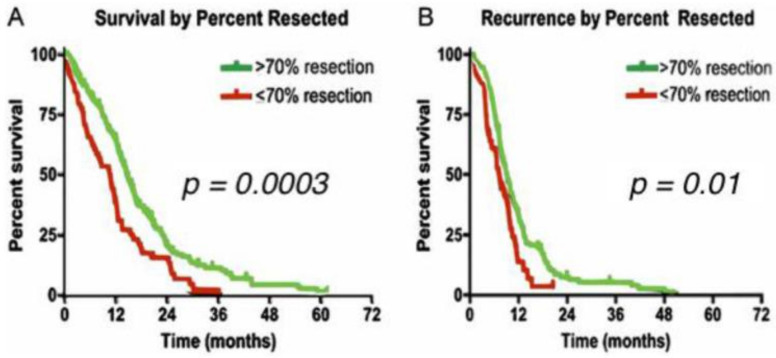
(**A**) Survival by extent of resection for high-grade gliomas. (**B**) Recurrence by extent of resection for high-grade gliomas. Greater than 70% extent of resection compared to less than 70% extent of resection [8].

The standard of care (SOC) for HGGs is maximally safe surgical resection, followed by concurrent radiation therapy (RT) and temozolomide (TMZ) for 6 weeks, then adjuvant TMZ for 6 months [4]. This SOC was established in 2005 after demonstrating a marginal increase in overall survival (OS) and progression-free survival at 6 months (PFS6) [5]. Surgically, the extent of resection (EOR) is the strongest predictor of OS [4]. HGGs cannot be treated by surgery alone because residual infiltrating glioma cells always extend well beyond the visible tumor mass, which is not seen in radiographic imaging [6]. The surgical goal is to resect the maximum amount of tumor possible while preserving neurological function. One study concluded that greater than 98% EOR improves OS significantly in GBM patients (13 vs. 8.8 months) [7]. However, more recent studies concluded that greater than 70% EOR also provides statistically significant improvement in OS (14.4 months vs. 10.5 months) and PFS6 (83.3% vs. 65.5%) in HGG patients (Figure 1) [6,8]. Most consider a gross total resection to be a resection of greater than 95% of the enhancing mass seen on MRI.

**Figure 2:**
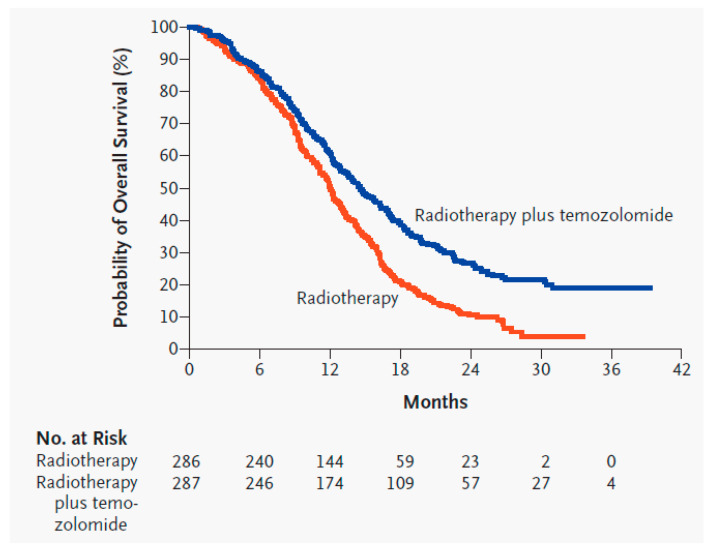
Kaplan–Meier estimates of overall survival of glioblastoma patients with radiotherapy alone compared to radiotherapy and temozolomide [5]. Used with permission.

**Figure 3:**
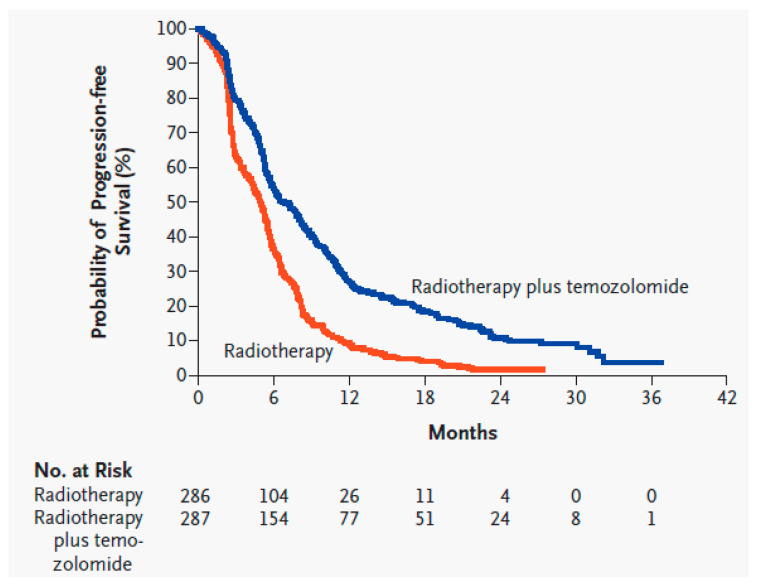
Kaplan–Meier estimates of progression-free survival of glioblastoma patients with radiotherapy alone compared to radiotherapy and temozolomide [5]. Used with permission.

Radiation is applied locally and kills tumor cells by causing nonspecific breaks in the DNA strands of rapidly dividing cells. Following surgery, RT is typically given for 6 weeks: 2 Gy per day, 5 days a week, for a total of 60 Gy [5]. Thus, 60 Gy in 30 fractions over 6 weeks was established as the SOC for HGGs in 1991 following the Bleehen et al. randomized controlled trial (RCT) which showed statistical significance in OS compared to 45 Gy in 20 fractions over 4 weeks (12 vs. 9 months) [9]. Common toxicities from 60 Gy include moderate-to-severe fatigue (26%) and thromboembolic events (5.5%) [5]. RT past 60 Gy has not been shown to increase survival [10,11]. Reducing doses or fractions has shown benefit in patients who are poor candidates for the full dose, such as the elderly or those with a poor Karnofsky Performance Status (KPS) [12,13,14]. Therefore, 40 Gy in 15 fractions is an accepted alternative in the SOC for patients 65 years of age or older [14,15]. During the same time period, TMZ is administered orally: 75 mg/m^2^ of body surface area per day, 7 days a week. TMZ is a nonspecific alkylating agent that causes mismatch repair in DNA by methylation at the O^6^ position of guanine, which triggers apoptosis in tumor cells [5,16]. Common toxicities include thrombocytopenia (12%), leukopenia (7%), and neutropenia (7%) [5]. Following 6 weeks of RT and TMZ, adjuvant TMZ is administered for six 28 day cycles: 150–200 mg/m^2^ of body surface area per day, 5 days a week. Adjuvant TMZ administration past six cycles has not been shown to increase survival [17]. Before this SOC was established, GBM patients typically lived for about a year after diagnosis, and no adjuvant chemotherapy had demonstrated significant survival benefits compared with radiation alone [5]. In 2005, the Stupp et al. RCT on GBM patients concluded that RT plus concurrent TMZ compared to RT alone significantly improved OS (14.6 vs. 12.1 months) and PFS6 (53.9% vs. 36.4%) [5], and later trials/meta-analyses have supported those results (Figure 2 and Figure 3, Table 2 and Table 3) [4,18,19,20,21].

### O^6^-Methylguanine DNA Methyltransferase Activity Predicts SOC Treatment Response

**Figure 4:**
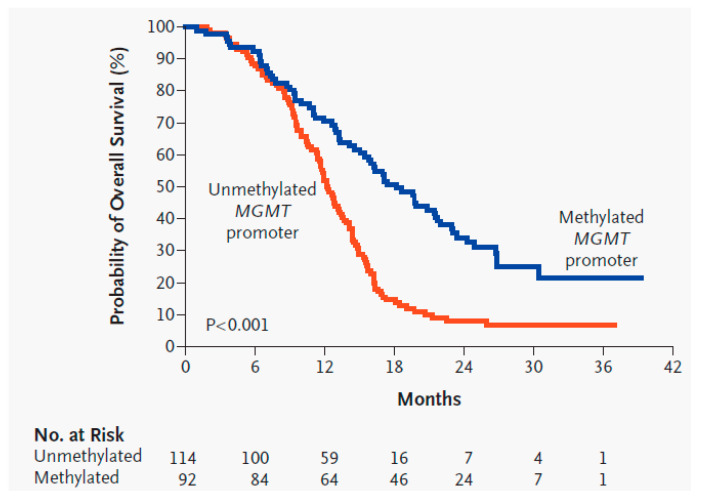
Kaplan–Meier estimates of overall survival of glioblastoma patients with a methylated MGMT promoter compared to an unmethylated MGMT promoter [16]. Used with permission.

O^6^-methylguanine DNA methyltransferase (MGMT) is a DNA-repair enzyme that repairs DNA adducts at the O^6^ position of guanine, which prevents the death of tumor cells via alkylation [22,23]. The gene that encodes MGMT is located on chromosome 10q26. Epigenetic silencing of the MGMT gene by promoter methylation characteristically shows decreased MGMT and DNA-repair activity. In 2005, the Hegi et al. RCT on GBM patients treated with RT and TMZ concluded that patients whose tumor contained a methylated MGMT promoter compared to an unmethylated MGMT promoter had significantly improved OS (21.7 months vs. 12.7 months) and PFS6 (68.9% vs. 40%) (Figure 4) [16]. MGMT status is often used to predict response to TMZ for patient counseling and to help with treatment choices. For example, in the elderly or those with a poor KPS, MGMT status may be used to choose between TMZ and RT when patients cannot tolerate both [24].

## 3. FDA-Approved Therapies

Outside of TMZ, there are four drugs and one device FDA-approved for the treatment of high-grade gliomas: lomustine, intravenous carmustine, carmustine wafer implants, bevacizumab, and tumor treatment fields (Table 4) [25,26,27]. These treatments are mainly FDA-approved for recurrent HGGs; only TMZ, tumor treatment fields, and carmustine wafer implants are approved for new diagnoses. All HGGs will progress [28]. There is no established SOC for these recurrences. Repeat surgery is an option that may provide symptom relief, but has not been concluded to increase OS [29,30]. A systematic review and meta-analysis suggested that re-irradiation has had positive effects, but requires randomized controlled trials to prove efficacy [31].

### 3.1. Lomustine

Lomustine (CCNU; chloroethylcyclohexylnitrosourea) was approved by the FDA to treat HGGs in 1976 [25]. In 1979, the Hochberg et al. RCT reported a median OS of 11.5 months [32]. CCNU or carmustine (BCNU; bis-chloroethylnitrosourea), alone or in combination with other chemotherapeutic drugs, was the SOC following surgery and/or radiation prior to the early 21st century. Currently, CCNU is solely approved for recurrent HGGs. CCNU is a nonspecific alkylating agent that causes crosslinking of DNA and RNA in dividing cells, which triggers cell death in tumor cells [37]. CCNU is administered orally at a dose of 80–110 mg/m^2^ once every 6 weeks [38]. Common toxicities are frequent and include hematologic toxicity (49.7%) [39]. CCNU is the SOC for recurrent GBM in Europe, and is often used as a control arm in recurrent GBM trials [40]. The efficacy of CCNU for GBM is improved in patients with MGMT promoter methylation [40]. In 2017, the Wick et al. RCT concluded that CCNU in combination with bevacizumab did not provide a survival advantage compared to CCNU alone (OS: 9.1 vs. 8.6 months; PFS: 4.2 vs. 1.5 months) [39]. CCNU is considered the key factor in the PCV regimen (P: procarbazine, C: lomustine, V: vincristine) which has been approved by the FDA as a regimen for HGGs [41]. In 2010, the Brada et al. RCT compared PCV to TMZ in patients with recurrent HGGs; they concluded PCV did not differ significantly in OS (6.7 vs. 7.2 months), PFS (3.6 vs. 4.7 months), quality of life, or adverse events [42]. Another study reported minimal toxicity and no observed adverse events from PCV treatment [43]. The PCV regimen is less commonly prescribed than CCNU alone.

### 3.2. Carmustine

Carmustine (BCNU; bis-chloroethylnitrosourea) was approved by the FDA to treat HGGs in 1977 [25]. In 1978, the Walker et al. RCT reported a median OS of 11.75 months [33]. Currently, BCNU is only FDA-approved to treat recurrent GBM. Similar to CCNU, BCNU is a nonspecific alkylating agent that causes crosslinking of DNA and RNA. It also binds to and modifies glutathione reductase, which leads to cell death in tumor cells [44]. BCNU is administered intravenously at a dose of 150–200 mg/m^2^ once every 6 weeks [45]. Common toxicities include pulmonary toxicity (<30%), ocular toxicity (>10%), and bone marrow suppression (>10%) [46]. High levels of toxicity and more effective treatments have resulted in IV BCNU being used less for HGGs than other therapies.

### 3.3. Carmustine Wafer Implants

Carmustine wafer implants were approved by the FDA for recurrent HGGs in 1996 and new HGGs in 2003 [25]. These biodegradable polymer wafers are about 1.45 cm in diameter, 1 mm thick, and contain 7.7 mg of BCNU per wafer [47]. The recommended dose is 8 wafers: 61.6 mg in total [48]. These wafers are applied directly to the tumor resection cavity intraoperatively for better locoregional treatment, increasing efficacy, and decreasing toxicity [49]. Common toxicities can be severe and include wound healing complications (12%), intracranial infection (1–10%), and cerebral edema (1–10%) [46]. In 2003, the Westphal et al. RCT on HGG patients concluded that BCNU wafers significantly improved OS (13.9 vs. 11.6 months), but not PFS (5.9 vs. 5.9 months) [34]. In 2008, one study reviewed treated GBM patients over a 10-year period: the median OS was 13.5 months after primary resection, only slightly better than the Stupp et al. [5] control group [50]. MGMT promoter methylation is correlated with increased OS in patients over 70 (13.5 vs. 7.6 months) treated with BCNU wafers [51]. One study reviewed 10 years (1997–2006) of patients treated with BCNU wafers and TMZ; they reported a very strong median OS of 20.7 months and PFS6 of 93%, much better than the Stupp et al. [5] results with TMZ [52]. In a review of 19 studies on 795 BCNU wafer patients, Bregy et al. reported a mean OS of 16.2 months and a staggering complication rate of 42.7%, prompting them to recommend not using the agent [53]. Furthermore, BCNU wafers are quite expensive. Despite demonstrated efficacy, BCNU wafer use has not become SOC. This may be due to its very high cost, reported high complication rates, and challenges of directly handling the agent by operating room staff.

### 3.4. Bevacizumab

Bevacizumab (BVZ) was approved by the FDA to treat recurrent GBM in 2009 [35]. BVZ is a targeted therapeutic antibody that binds and inhibits the vascular endothelial growth factor (VEGF) protein in tumor cells. Malignant gliomas characteristically have robust neovascularity, likely due to overexpression of VEGF and other proangiogenic factors. BVZ is often used to inhibit VEGF and attempts to prevent tumor angiogenesis, which decreases tumor vasculature and blood supply, slowing the spread of tumor cells [54]. BVZ is administered intravenously at a dose of 10 mg/kg once every 2 weeks [55]. Although BVZ is well-tolerated, impaired VEGF function is linked to multiple common toxicities: hypertension (5.5–11.4%), thromboembolic events (3.2–11.9%), gastrointestinal perforation (1.5–5.4%), cerebral bleeding (2–5.3%), wound healing complications (0.8–3.3%), and proteinuria (2.7–11.4%) [56]. BVZ is FDA-approved as a monotherapy and in combination with irinotecan [35,57]. A number of cytotoxic agents such as etoposide and carboplatin when paired with bevacizumab showed benefits in clinical trials for recurrent GBM, but are not officially FDA-approved [58,59]. In 2014, the Gilbert et al. RCT compared BVZ and TMZ with TMZ alone for newly diagnosed GBM patients: BVZ did not increase OS (15.7 vs. 16.1 months), did increase PFS (10.7 vs. 7.3 months), but resulted in increased side effects, increased symptom burden, decreased neurocognitive function, and worse quality of life [60]. In 2018, the Ameratunga et al. study of 11 anti-angiogenic therapy HGG RCTs concluded that anti-angiogenic treatment (often BVZ) paired with chemotherapy did not significantly improve OS or PFS compared to chemotherapy alone [61].

Despite the lack of efficacy in more current trials as a primary treatment, BVZ continues to be used currently to treat symptomatic edema and radiation necrosis. Primary brain tumors often present with peritumoral brain edema (PBTE) which increases local mass effect and intracranial pressure, causing a variety of neurological symptoms [62]. In 2017, the Meng et al. study reported that BVZ improved clinical symptoms the day after treatment in 84.74% of patients with refractory brain edema [63]. By reducing edema, BVZ also allows the reduction of steroid medications and their side effects. Brain radiation necrosis (BRN) is the death of healthy brain tissue caused by RT occurring in 2.5–24% of patients that receive radiation [64,65]. Fluid-attenuated inversion recovery (FLAIR) abnormalities and T1-weighted post-Gd-contrast abnormalities are MRI diagnostic markers of BRN. In 2007, Gonzalez et al. were the first to treat BRN with BVZ; FLAIR abnormalities and T1-weighted post-Gd-contrast abnormalities significantly decreased in 8 of 8 patients [66]. Later studies have supported those results; however, the irreversibility and recurrent nature of BRN warrants further studies [67]. It is important to note that BRN is difficult to detect from MRI and there currently is not a way to reliably differentiate recurrence from pseudoprogression. However, there are adjunctive tools that when reviewed in total may help guide clinicians. MRI spectroscopy and MRI perfusion have often been used to help differentiate these entities. Fluorodeoxyglucose positron emission tomography (FDG-PET) and 18-fluoride-fluoro-ethyl-tyrosine positron emission tomography (FET-PET) have also been useful in determining BRN.

### 3.5. Tumor Treatment Fields

**Figure 5:**
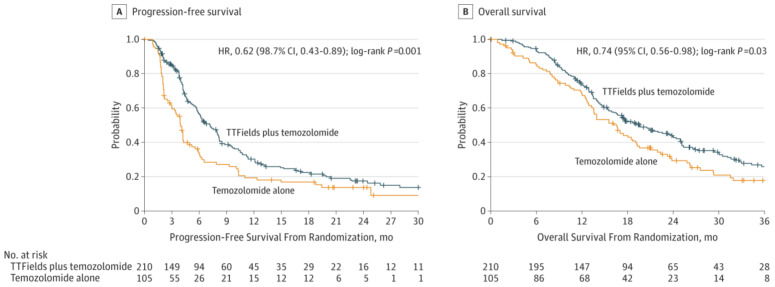
(**A**) Progression-free survival of glioblastoma patients with tumor-treating fields plus temozolomide compared to temozolomide alone. (**B**) Overall survival of glioblastoma patients with tumor-treating fields plus temozolomide compared to temozolomide alone [36]. Used with permission.

Tumor treatment fields (TTFields) was approved by the FDA for recurrent GBM in 2011 and newly diagnosed GBM in 2015 [25]. It is a portable device applied to the shaved scalp for more than 18 h a day and a minimum of 4 weeks. TTFields deliver low-intensity (1–3 V/cm), intermediate-frequency (200 kHz) alternating electric fields that disrupt mitosis in tumor cells. Common toxicities include skin toxicity (43%) and seizures (7%) [36]. TTFields is used in combination with TMZ. In 2015, the Stupp et al. RCT on GBM patients concluded that TTFields plus TMZ compared to TMZ alone significantly improved OS (20.5 vs. 15.6 months) and PFS (7.1 vs. 4.2 months) (Figure 5) [36]. Later studies supported those results and showed an improved PFS6 (56% vs. 37%) [68,69]. Despite its efficacy, there is only moderate acceptance among patients and providers [70]. After the Stupp et al. [36] RCT, many argued that TTFields should become a part of the SOC. A roundtable discussion was held among some of the leading brain cancer experts in the world. They decided that TTFields would not be added to the SOC because of marginal survival benefits, expensive costs, and inconvenience for patients [71].

### 3.6. 5-Aminolevulinic Acid

5-aminolevulinic acid (5-ALA) is an intraoperative imaging agent that was approved by the FDA in 2017, and allows for intraoperative visualization of malignant glioma tissue via fluorescence-guided surgery (FGS) [25]. 5-ALA is administered orally prior to surgery. 5-ALA metabolizes into protoporphyrin IX, causing the fluorescence in tumor cells [72]. In 2006, the Stummer et al. RCT on HGG patients concluded that the use of 5-ALA compared to white light significantly increased complete contrast-enhancing tumor resection (65% vs. 36%) and PFS6 (41% vs. 21.1%) [73]. Additional studies have suggested more complete resections and better patient outcomes from 5-ALA FGS than conventional white light microsurgery [25,72]. A short half-life of approximately 3 h and over-resection are potential adverse effects of 5-ALA FGS. 5-ALA can potentially demonstrate some fluorescence in adjacent non-tumorous brain, so neurosurgeons must keep this in mind. On the other hand, there are robust data to suggest that supra marginal resection (removal of at least 1 cm of brain tissue surrounding the contrast enhancement) may add survival benefit [74]. It is critical for the neurosurgeon to focus on maximally safe resections, but in some situations there may be a role for supramarginal resection. This remains to be adequately proven and is currently not a standard of care for surgery.

Currently, 5-ALA FGS is not widely used and although it does not directly kill tumor cells, it may help some achieve more EOR, which theoretically should improve OS. However, data are not conclusive if the use of 5-ALA clearly improves OS. It remains one of numerous surgical adjuncts that neurosurgeons may use to help in the operating room.

## 4. Future Directions

Currently, the majority of FDA-approved therapies for HGGs are nonspecific agents and provide marginal survival benefit. Currently, there have not been transformational additions to the treatment of HGG beyond surgical resection. Patients who are not surgical candidates have no great options. The turn of the century did bring hope. The new genomic era has made it possible to map individual patient glioma genomes and describe the molecular features of a glioma tumor in granular detail. A rapidly growing library of specific targeting agents in the field of oncology has the potential to demonstrate better efficacy and lower toxicity. A multitude of specific agents are available to test on HGGs, but the challenge of few patients with limited survival time (12–18 months) for testing remains. The rareness of the disease makes the study patient population limited. Most clinical trials limit study participants by factors such as age, KPS, molecular profile, and concomitant trials. Between 2005 and 2016, only 8–11% of GBM patients enrolled in clinical trials. The median GBM clinical trial duration was between 3 and 4 years. During the same time period, only 1 of 8 GBM completed phase III clinical trials concluded with efficacy, even though 58% were supported by phase II data [75]. Ruling out ineffective therapies sooner (e.g., via adaptive clinical trial) and better recruitment of participants (e.g., less restrictive recruitment) to clinical trials could be beneficial in finding a more efficacious treatment for GBM. The speed of SARS-COV-2 pharmaceutical and vaccination trials amidst the COVID-19 pandemic proves that we can do better.

A number of therapies are being studied and tested to treat HGGs. Using a recombinant poliovirus as an oncolytic viral therapy has shown potential for efficacy. In 2018, the Desjardins et al. RCT on recurrent GBM patients concluded that the intratumoral delivery of the recombinant nonpathogenic polio–rhinovirus chimera (PVSRIPO) provided an improved OS at 24 and 36 months compared to historical controls [76]. PVSRIPO patients had an OS of 21% at both 24 and 36 months compared to a continued decline from historical controls [76]. The plateau suggests a potential cure for these types of patients. The PVSRIPO therapy is not currently FDA-approved for HGGs, but may be beneficial for long-term survival in the future. Larger multicenter studies are needed to further demonstrate clinical significance.

**Figure 6:**
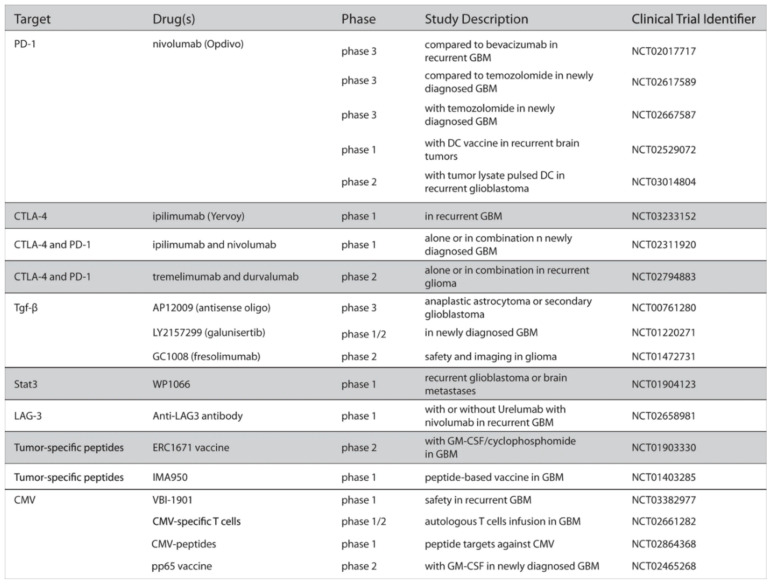
Therapies in clinical trials for high-grade gliomas [77]. Used with permission.

Nivolumab (NIVO) is an example of immune checkpoint blockade and is a PD-1 inhibitor. The inhibition of PD-1 enables T-cells to attack cancer cells. NIVO is in multiple clinical trials for both new and recurrent GBM (Figure 6) [77]. Ipilimumab and tremelimumab are similar monoclonal antibodies that prevent the inhibition of T-cell-mediated immune responses [78]. Transforming growth factor-beta (TGF-β) is a cytokine involved in the proliferation of cells. Studies have discovered overexpressed TGF-β in malignant glioma tissue [79]. There are multiple TGF-β targeted medications undergoing clinical trials for gliomas (Figure 6) [77]. Peptide-based vaccines are a form of immunotherapy being tested in trial on HGG patients. The vaccines are usually derived from cancer cells then injected to generate an antitumor response via activated lymphocytes [80].

## 5. Conclusions

The current SOC for HGGs is maximally safe surgical resection, followed by concurrent RT and TMZ for 6 weeks, then adjuvant TMZ for 6 months. This SOC was established in 2005 after demonstrating a marginal increase in OS and PFS6. CCNU, IV BCNU, BCNU wafer implants, BVZ, and TTFields are FDA-approved therapies now mainly used to treat recurrent HGGs and symptoms. Only TMZ, TTFields, and BCNU wafer implants are approved for new diagnoses. TTFields is the only treatment that has been shown to improve OS and PFS6 in comparison to the current SOC. Current FDA-approved therapies provide marginal survival benefit, and a rapidly growing library of specific targeting agents in the field of oncology has potential to demonstrate better efficacy and lower toxicity for HGGs.

## Figures and Tables

**Table 1:** 5-year survival rates for high-grade gliomas by age [1,2].

Type of Tumor (WHO Grade)	5-Year Relative Survival Rate		
	Age		
	20–44	45–54	55–64
Anaplastic astrocytoma (III)	58%	29%	15%
Glioblastoma (IV)	22%	9%	6%
Anaplastic oligodendroglioma (III)	76%	67%	45%

**Table 2:** Overall survival of glioblastoma patients with radiation therapy alone compared to radiation therapy plus temozolomide [5].

Variable	RT Alone (n = 286)	RT Plus TMZ (n = 287)
Median OS (months)	12.1 months	14.6 months
OS at 6 months (%)	84.20%	86.30%
OS at 12 months (%)	50.60%	61.10%
OS at 18 months (%)	20.90%	39.40%
OS at 24 months (%)	10.40%	26.50%

**Table 3:** Progression-free survival (PFS) of glioblastoma patients with radiation therapy alone compared to radiation therapy plus temozolomide [5].

Variable	RT Alone (n = 286)	RT Plus TMZ (n = 287)
Median PFS (months)	5 months	6.9 months
PFS at 6 months (%)	36.40%	53.90%
PFS at 12 months (%)	9.10%	26.90%
PFS at 18 months (%)	3.90%	18.40%
PFS at 24 months (%)	1.50%	10.70%

**Table 4:** List and information on FDA-approved therapies for high-grade gliomas.

FDA-Approved Therapy	Year Approved	Randomized Controlled Trial	Approved for	Mechanism	Application	Dosage	Common Toxicities	Overall Survival	Progression-Free Survival at 6 Months	Other Notes
Lomustine (CCNU)	1976	Hochberg et al., 1979 [32]	Recurrent HGG	Nonspecific alkylating agent that causes crosslinking of DNA and RNA in dividing cells triggering cell death	Oral	80–110 mg/m^2^ every 6 weeks	Hematologic toxicity (49.7%)	11.5 months	Unknown	No benefit compared to RT alone
Carmustine (BCNU)	1977	Walker et al., 1978 [33]	Recurrent HGG	Nonspecific alkylating agent that causes crosslinking of DNA and RNA in dividing cells; also binds to and modifies glutathione reductase	IV	150–200 mg/m^2^ every 6 weeks	Pulmonary toxicity (<30%), ocular toxicity (>10%) and bone marrow suppression (>10%)	11.75 months	Unknown	No benefit compared to RT alone
Carmustine wafer implants (BCNU wafers)	1996 & 2003	Westphal et al., 2003 [34]	Recurrent and new HGG	Nonspecific alkylating agent that causes crosslinking of DNA and RNA in dividing cells; also binds to and modifies glutathione reductase	Directly applied during surgery	8 wafers: 61.6 mg	Wound healing complications (12%), intracranial infection (1–10%), and cerebral edema (1–10%)	13.9 months	Unknown	High complication rate (42.7%) and expensive
Temozolomide (TMZ)	2005	Stupp et al., 2005 [5]	All HGGs (SOC)	Nonspecific alkylating agent that causes mismatch repair in DNA by methylation at the O^6^ position of guanine	Oral	75 mg/m^2^ per day with RT, 150–200 mg/m^2^ per day	Hematologic toxicity (16%): thrombocytopenia (12%), leukopenia (7%), and neutropenia (7%)	14.6–16.1 months	53.90%	Standard of Care
Bevacizumab (BVZ)	2009	Cohen et al., 2009 [35]	Recurrent HGG	Targeted therapeutic antibody that binds and inhibits VEGF protein in tumor cells	IV	10 mg/kg every 2 weeks	Hypertension (5.5–11.4%), thromboembolic events (3.2–11.9%), gastrointestinal perforation (1.5–5.4%), cerebral bleeding (2–5.3%), wound healing complications (0.8–3.3%), and proteinuria (2.7–11.4%)	9.3 months (recurrent)	36% (recurrent)	Used to treat symptomatic edema and radiation necrosis
Optune device (TTFields)	2011 & 2015	Stupp et al., 2015 [36]	Recurrent and new HGG	Low-intensity (1–3 V/cm), intermediate-frequency (200 kHz) alternating electric fields that disrupt mitosis in tumor cells	Portal device, electrodes on scalp	Greater than 18 h a day for >4 weeks	Skin toxicity (43%) and seizures (7%)	20.5–20.9 months	56%	Not SOC because of marginal survival benefits, expensive costs, and inconvenience for patients

## Data Availability

Not applicable.
